# Spontaneous corneal melting in pregnancy: a case report

**DOI:** 10.1186/1752-1947-1-143

**Published:** 2007-11-22

**Authors:** Sudesh K Arya, Archana Malik, Sonika Gupta, Hemlata Gupta, Sunandan Sood

**Affiliations:** 1 Department of Ophthalmology, Government Medical College and Hospital, Chandigarh, India

## Abstract

**Background:**

To report a case of spontaneous corneal melting in pregnancy. We reviewed the literature on corneal melting and the effect of pregnancy on cornea and collagen containing tissues.

**Case presentation:**

A 29-year-old woman who underwent radial keratotomy in both eyes followed by trabeculectomy in her left eye developed corneal melting in the same eye, in her seventh month of pregnancy. Despite screening, no infectious or immune mediated condition could be identified. She was managed conservatively with cyanoacrylate glue, bandage contact lens, lubricants and antibiotics.

**Conclusion:**

It may not always be possible to find the underlying cause of corneal melting but the more common underlying causes should be ruled out by proper investigations. Pregnancy with its host of hormonal changes could potentially have some effect on corneal collagen leading to corneal melting in compromised corneas.

## Background

A large number of hormonal, metabolic, immunologic, hematologic and cardiovascular changes occur during pregnancy which affects all the tissues including the eye. The effects of pregnancy on the eye are described in three categories[1]. *Nonpathological physiological *changes like corneal edema with increase in corneal thickness and curvature, decreased corneal sensitivity, increased aqueous outflow facility and changes in visual field[1]. *Pathological conditions *reported to develop during pregnancy include central serous retinopathy, hypertensive and vascular disorders and uveal melanoma[1]. Pregnancy also affects *pre-existing ocular conditions *such as diabetic retinopathy, tumors and a variety of immunological disorders and can have beneficial effects on certain pre-existing conditions like glaucoma[1]. We did not find any reported case of spontaneous corneal melting with perforation in pregnancy. We report a case of a pregnant woman who developed spontaneous corneal melting in her 7^th ^month of pregnancy.

## Case presentation

**Figure 1:**
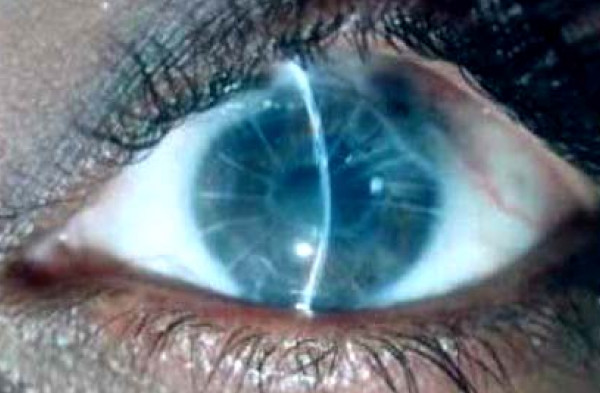
Left eye with flat anterior chamber (before cyclocryotherapy).

**Figure 2:**
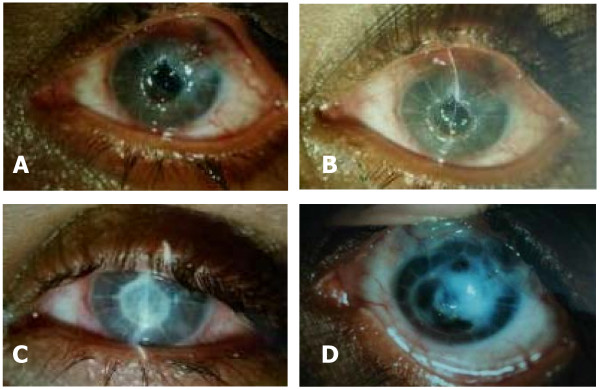
A and B: Left eye showing central corneal melting with perforation inferiorly. C: Cyanoacrylate glue and bandage contact lens applied. D: Healed stage with leucomatous corneal opacity.

A 29-year old woman who was in her seventh month of pregnancy presented with chief complaints of sudden onset of pain and watering in her left eye of 4 days duration. Her past history revealed the onset of myopia at the age of 12 years which gradually increased and stabilized by 18 years of age. At the age of 20 years she underwent radial keratotomy in both eyes for myopic correction. She developed secondary angle closure glaucoma in left eye following radial keratotomy (probably there was microperforation at the time of radial keratotomy, leading to shallow anterior chamber with formation of peripheral anterior synechiae leading to raised IOP) for which she underwent glaucoma filtering surgery without Mitomycin C elsewhere 8 months later. Records revealed IOP of less than 21 mmHg till few months postoperatively. A year later, she presented to us with a painful eye which on examination revealed flat anterior chamber in the periphery and very shallow centrally (Fig. 1). The intraocular pressure as measured by applanation tonometer was 40 mmHg and her vision was light perception with inaccurate projection of rays. Posterior segment examination revealed glaucomatous optic atrophy in left eye. Cyclocryotherapy was done after which the intraocular pressure came back to normal. She remained asymptomatic for the next few years, during which she conceived. The first and second trimesters were uneventful, but during the seventh month of pregnancy she presented with sudden onset of pain, redness and watering but no discharge in the left eye. Examination revealed central corneal melting measuring 6 × 6 mm and an area of corneal perforation inferiorly of approximately 2 × 3 mm. There was mild corneal edema without any evidence of active infiltration and rest of the cornea was clear. Corneal sensations were normal and equally brisk in both eyes. On slit lamp examination, a very thin layer of posterior corneal stroma could be seen in the area of melting. The anterior chamber was flat (Fig. 2a, b). Intraocular pressure was low digitally. Gram and KOH stain and culture on Blood and Sabouraud's dextrose agar were negative. There was no history of trauma or any other systemic illness and the patient did not exhibit any clinical features of systemic vasculitis or autoimmune condition. Rheumatoid factor, antinuclear antibody, anti-cytoplasmic and anti-DNA antibodies were negative. Patient had not used any topical drops during the intervening period. The vision in her right eye was 6/6 with -1.50 Diopters and the examination was essentially unremarkable. The patient being in the late stage of pregnancy and having a poor visual prognosis, conservative management was planned. Cyanoacrylate glue and bandage contact lens were applied and topical antibiotics, cycloplegics and lubricating drops were prescribed (Fig. 2c). After 3 weeks of conservative treatment, corneal edema decreased and corneal perforation gradually healed. Anterior chamber remained flat, although the intraocular pressure was normal digitally. She was kept on regular follow up and on her last visit; leucomatous corneal opacity was seen at the involved site (Fig. 2d).

## Discussion and conclusion

Corneoscleral melting is commonly seen in immune mediated conditions, most common being rheumatoid arthritis[2]. Infections, chemical burns[3], recti surgery[4], use of topical steroids, non steroidal anti-inflammatory drugs and non-absorbable sutures[5-7] have been reported as causes of corneal melting and necrotizing scleritis. Rare causes of corneal melting that have been reported in literature are paraneoplastic pemphigus[8], pyoderma gangrenosum[9], Vogt-Koyanagi-Harada syndrome and psoriasis[10]. In our case, despite screening; we couldn't find evidence of any of the above.

During pregnancy there are host of hormonal changes, which have different effects on various parts of the body. One of the important hormones produced during pregnancy is relaxin, which is thought to play an important regulatory role in collagen remodeling during gestation. It is a positive regulator of matrix metalloproteinase which has collagenolytic activity. It has differential effect on various collagen containing organs. In various animal models it has been observed that there is a change in the type and content of collagen in pubic symphysis, uterus and cervix [11], cartilage [12], and aorta [13]. Striae gravidarum, seen in pregnancy results in tearing of collagen matrix of dermis and weakness of elastic fibers [14].

It has also been documented that hormonal changes during pregnancy induce corneal edema. Corneal sensitivity has been seen to decrease in pregnant women, with maximum changes in the later half of the pregnancy, which returns to normal by 6 to 8 weeks after delivery[1]. Decline in corneal sensitivity is primarily attributed to corneal edema. On Medline search we found one case report of corneal perforation (not melting) in pregnancy with pre-existing keratoconus. Lahoud et al (1987) reported a case of 24 year old female who had history of bilateral keratoconus and presented with marked corneal edema and perforation in her eighth month of pregnancy and was eventually treated by penetrating keratoplasty [15].

We hypothesize that relaxin, by virtue of its differential effect, probably acts on corneal collagen as well and may exhibit collagenolytic property. These changes may not be of major concern in a healthy cornea but in compromised corneas they may lead to corneal melting and devastating complications, as seen in our case. Pregnancy as a cause of corneal melting has not been earlier documented, but after extensive investigations and detailed history in our case we could not find any other cause. Further studies are required to evaluate this hypothesis.

The case demonstrates that in high risk females who have compromised corneas, the physiological changes during pregnancy may contribute to development of devastating complications. Hence, such patients should be carefully monitored during pregnancy atleast once in each trimester so that prophylactic treatment can be started at the earliest.

## Abbreviations

**IOP- **Intraocular pressure

## Competing interests

The author(s) declare that they have no competing interests.

## Authors' contributions

**SKA **was the primary treating surgeon and managed the case. He was also involved in revising the manuscript critically and gave important intellectual contribution.

**AM **was involved in management of the case. She wrote the first draft, reviewed the literature and was involved in final preparation and submission of manuscript.

**SG, HG, SS **were involved in revising the manuscript critically and gave important intellectual contribution at the time of preparation of the manuscript

## Consent

Informed and written consent of the patient was taken for publishing this case report and utilizing the photographs for publication.
